# Prevalence of Maternal Anemia in A Tertiary Care Hospital in Western Nepal

**DOI:** 10.31729/jnma.4546

**Published:** 2019-08-31

**Authors:** Anita Lamichhane, Sharmila Gurung, Kiran Panthee, Deekshya Shrestha

**Affiliations:** 1 Department of Pediatrics, Lumbini Medical College, Pravas, Palpa, Nepal; 2 Department of Forensic Medicine, Devdaha Medical College, Bhaluhi, Rupandehi, Nepal; 3 Department of Pediatrics, Devdaha Medical College, Bhaluhi, Rupandehi, Nepal; 4 Department of Obstetrics & Gynaecology, Devdaha Medical College, Bhaluhi, Rupandehi, Nepal

**Keywords:** *maternal anemia*, *pregnancy*, *safe motherhood*

## Abstract

**Introduction:**

Maternal anemia is a common problem in developing countries like Nepal accounting for around 30-50% of women becoming anemic during pregnancy. The present study aims to find out the prevalence of maternal anemia in a tertiary care hospital in Western Nepal.

**Methods:**

A descriptive cross-sectional study was carried out at Devdaha Medical College, Bhaluhi, Rupandehi, Nepal from October 2018 to May 2019 after taking ethical approval from the institutional review committee with the approval number: 012/2018. Three eighty three samples were taken and convenient sampling was done to reach the sample size. Data were collected from the study population after taking consent and entered in a predesigned proforma. It was then entered in an SPSS; point estimate at 95% CI was calculated along with frequency and proportion for binary data.

**Results:**

During the study period, out of 383 mothers, 230 (60.2%) mothers were anemic at 95% CI (10.6-10.8%); of which 172 (74.8%) were moderately anemic while 58 (25.2%) were mild anemic. The mean maternal Haemoglobin was 9.5±1.76SD. The mean maternal age was 24.24±3.26 SD; mean gestational age at the time of delivery was 36.08±1.77 SD.

**Conclusions:**

The prevalence of maternal anemia in this study is found to be higher than the national data which implies that maternal anemia is still a public health issue which needs to be addressed in spite of safe motherhood program launched by the government of Nepal.

## INTRODUCTION

Maternal anemia, in developing countries like Nepal is the most common medical condition accounting for around 30-50% of women becoming anemic during pregnancy.^1^ WHO defines anemia as haemoglobin<11 grams% in pregnancy, mild anemia (10–10.9 g/dL), moderate anemia (7–9.9 g/dL) and severe anemia (<7 g/dL).^2,3^ Globally, about 38.2% of pregnant mothers are anemic.^4^ Nepal Demographic Health Survey (2011) shows the prevalence rate of anemia in pregnancy to be 48%.^5^

Maternal anemia is associated with Post-Partum Haemorrhage (PPH), Low Birth Weight (LBW) babies, prematurity, Small for Gestational Age (SGA) babies and perinatal death. This gestational outcome is considered as a major public health concern; it is more prevalent in countries with low financial resources.^6^ LBW (weight < 2500 grams) are more prone to infant morbidity and mortality.^7^

The objective of the study was to find the prevalence of maternal anemia in a tertiary care hospital in Western Nepal.

## METHODS

This hospital-based descriptive cross-sectional study was conducted from October 2018 to May 2019 at Devdaha Medical College, Bhaluhi, Rupandehi, Nepal after taking ethical approval from the institutional review committee (IRC) of the college. The present study included 383 pregnant mothers>18 years of age with ANC visits done at Devdaha Medical College with a singleton pregnancy using consecutive sampling method. Those pregnant women with multiple pregnancies, history of preterm delivery and with any obstetrical complications or medical illness except anemia were excluded from the study. We took a written consent from the mother to participate in the study for the mothers. A detailed history was taken from the mother during the presentation for delivery and the data of the mothers was entered in the predesigned proforma. The blood of the mothers were collected from the antecubital vein and stored in the EDTA containing vial and then analysed using the automated hematologic analyzer.

Maternal anemia was defined as Hb<11 g/L. All the information including gestational age at the time of delivery, mode of delivery, clinical signs and symptoms, indication for admission in NICU, maternal risk factors, were recorded in the predesigned proforma. The perinatal outcome was defined as the maternal and fetal consequences caused by maternal habits and pregnancy complications during labor and one hour after delivery. The maternal consequences included preterm delivery, prolonged labor and maternal mortality whereas fetal consequences included small for gestational age, low Apgar score, intrauterine growth retardation, and intrauterine death. The fetal outcomes were small for gestational age, congenital anomalies, low birth weight, stillbirth, respiratory distress syndrome, preterm babies, intrauterine growth retardation, low Apgar score less than 5 at 1 min and birth asphyxia.

Convenient sampling was done and the sample size was calculated using the formula,^8^

Sample size = Z^2^ ×pq/d^2^

    = 3.84 × 0.48×0.48/ (0.05)^2^

    = 383

Z =1.96 at 95% CI.
p= prevalence of maternal anemia, 48%^6^
q= 1-p
e= margin of error, 5%

The total sample size calculated was 383.

The mothers were followed until discharge or death. In case of death, the cause of mortality was recorded. Data were checked for any errors or inconsistencies, then entered in a Statistical Package for Social Sciences (SPSS), point estimate at 95% CI was calculated along with frequency and proportion for binary data.

## RESULTS

The prevalence of maternal anemia was found out to be 60.2% at 95% CI (10.6-10.8%) in our study. During the study period, 383 mothers and their newborn babies were evaluated. Out of them, 230 (60.2%) mothers were anemic; of which 172 (74.8%) were moderately anemic while 58 (25.2%) were mild anemic. No one was found to be severely anemic. The mean maternal Haemoglobin was 9.5±1.76 SD. There were 366 (95.5%) live-born, stillbirth 03 (0.8%) and IUFD 14 (3.7%) babies. The mean maternal age was 24.24±3.26 SD, (range=16-38 years, median=24.00); mean gestational age at the time of delivery was 36.08±1.77 SD. The mean birth weight of the baby was 2.35±0.374kg. Male: female ratio was 1.4:1.Perinatal mortality in our study was 44.3 per thousand population. In mothers with anemia, low birth weight was seen in 161 (42.0%) at 95% CI (2.312.38) cases and most of the mothers were from rural area 261 (68.1%). The demographic characteristics of our study population are depicted (Table 1).

**Table 1:** Showing demographic characteristics of the study population.

Characteristics	n (%)
**Outcome**	
Live born	366 (95.5)
Stillbirth	03 (0.8)
IUFD	14 (3.7)
**Mode of delivery**	
Normal delivery	183 (47.8)
Caesarean	171 (44.6)
Vacuum	29 (7.6)
**Place of residence**	
Urban	122 (31.9)
Rural	261 (68.1)
**Sex**	
Male	224 (54.49)
Female	158 (41.25)
Ambiguous genitalia	01 (0.26)
**Anemia in mother**	
Present	213 (55.61)
Absent	170 (44.39)
**Gestational age**	
Term delivery (≥37 weeks)	162 (42.3)
Preterm delivery (<37 weeks)	221 (57.7)
**Maternal death**	
Yes	01 (0.3)
No	382 (99.7)
**Early neonatal death**	
Yes	03 (0.8)
No	380 (99.2)
**Admission in NICU**	
No	318 (83)
Yes	65 (17)
**Anemia in baby**	
Yes	25 (6.5)
No	358 (93.5)
**Congenital anomalies**	
Absent	372 (97)
present	11 (2.9)

Among anemic mothers, anemia was found prevalent at 37 weeks of gestation following second most at 35 and 36 weeks respectively (Table 2).

**Table 2:** Showing the relationship between maternal anemia and gestational age at delivery.

Gestational	Anemia in mother	
age (weeks)	Yes n (%)	No n (%)
32	7 (1.82)	00 (0)
33	14 (3.7)	04 (2.6)
34	27 (7.0)	25 (16.3)
35	45 (11.7)	40 (26.1)
36	37 (9.7)	22 (14.4)
37	54 (14.1)	24 (15.7)
38	28 (7.3)	19 (12.4)
39	17 (4.4)	14 (9.2)
40	01 (0.3)	05 (3.3)
Total	230	153

One hundred and sixty one (42%) mothers with maternal anemia delivered low birth weight babies (Table 3).

**Table 3:** Showing the effect of maternal anemia on the weight of the baby.

Maternal anemia	Weight of the baby (n = 383)	
	< 2.5 kg (LBW)	≥2.5 kg
Present	161 (42.0)	68 (17.8)
Absent	74 (19.3)	80 (20.9)
Total	235 (61.3)	148 (38.7)

Perinatal outcome of babies born to anemic mothers showed live born in 224 (97.4%), low birth weight in 161 (42%), Preterm in 130 (56.5%), IUGR in 55 (23.9%), NICU admission in 39 (17%), anemia in baby in 14 (6.1%), IUFD in 10 (4.3%) and stillbirth in 2 (0.9%) (Table 4).

**Table 4:** Showing the perinatal outcome of maternal anemia.

Characteristics	Anemia in mother (n = 383)	
	Yes n (%)	No n (%)
Live born	224 (97.4)	142 (0.9)
IUFD	10 (4.3)	04 (2.6)
Still birth	02 (0.9)	01 (0.7)
IUGR	55 (23.9)	15 (9.8)
Low birth weight	161 (42.0)	74 (19.3)
Preterm	130 (56.5)	91 (59.5)
Admission in NICU	39 (17.0)	26 (17.0)
Anaemia in the baby	14 (6.1)	11 (7.2)

## DISCUSSION

During the study period, 383 mothers along with their newborns were included, of which 230 (60.2%) proportions of mothers were anemic. The prevalence of maternal anemia was found out to be 60.2% at 95% CI (10.6-10.8%) in our study. This is similar to a study reported from India 9 (60.38%) and 10 (62.3%) while it is in contrast to some studies done in other parts of Nepal which showed a low prevalence rate ranging from 42 to 48%.^11–12^ NDHS data set 2016 showed a prevalence rate of 40%.^13^ Our study revealed 74.8% of mothers were moderately anemic while 58 (25.2%) were mildly anemic. This indicates that the nutritional significance of nutrition is subordinate and awareness is made to the rectification of anemia in the pre-pregnancy period.

Our study showed mean maternal haemoglobin to be 9.5±1.76 gm/dL which is near to comparable from a study done in Nepal^14^ which showed the mean maternal haemoglobin concentration to be 11.14 ± 1.39 gm/dL.

Mean maternal age in our study was a 24.24±3.26 year which is similar to a study done by Timilsina et al.^15^ Mean gestational age at delivery was 36.08±1.77 which is similar to a study done in England^16^ and in Nepal.^17^

This study showed that the maximum number of mothers 261 (68.1%) were from the rural area. The percentage of low birth weight babies were more in mothers from a rural area 24.6% as compared to urban areas 13.5%. This was similar to a study done by Yadav et al where 84% mothers residing in rural areas of terrain region of Nepal had a proportion of LBW in rural 21.71% and urban 20.83% areas.^18^

Our study showed the mean birth weight to be 2.35±0.37 kg. Low Birth Weight was seen in 161 (42.0%) cases of anemic mothers which is very high and is in contrary to a study done by Acharya et al which showed to be only 19.4%^19^ and 9.8% in a study done in Nepal.^18^ Another study showed the prevalence rate of 23.6%.^20^ In the present study out of the total low birth weight babies, 56.2% were males and 43.8% were female which is similar to a study done by Ahmed et al^20^ while it is in contrast to a study done by Hussain et al in Pakistan which showed the incidence of male 45.2% and female 55%.^21^

Our study showed mothers having preterm delivery were 23.4% and the proportion of low birth weight was high. A study from Karnataka showed the preterm birth was 38%.^22^ Perinatal mortality in our study was 44.3 per thousand population which is similar to a study done 42%.^23^

## CONCLUSIONS

The high prevalence rate of maternal anemia in this study implies that maternal anemia is still a public health issue which needs to be addressed in spite of the safe motherhood program launched by the government of Nepal. Low maternal haemoglobin levels are associated with increased risk of stillbirth and IUD, and LBW babies. The prevalence of low birth weight was found to be significantly high among institutional deliveries of this region of the country.

## Conflict of Interest:

**None.**
